# Ultrasonic Retinal Neuromodulation and Acoustic Retinal Prosthesis

**DOI:** 10.3390/mi11100929

**Published:** 2020-10-13

**Authors:** Pei-An Lo, Kyana Huang, Qifa Zhou, Mark S. Humayun, Lan Yue

**Affiliations:** 1 Roski Eye Institute, University of Southern California, Los Angeles, CA 90033, USA; peianlo@usc.edu (P.-A.L.); kyanahua@usc.edu (K.H.); qifazhou@usc.edu (Q.Z.); humayun@med.usc.edu (M.S.H.); 2 Ginsburg Institute for Biomedical Therapeutics, University of Southern California, Los Angeles, CA 90033, USA

**Keywords:** neuromodulation, neurostimulation, ultrasound, retina, visual restoration

## Abstract

Ultrasound is an emerging method for non-invasive neuromodulation. Studies in the past have demonstrated that ultrasound can reversibly activate and inhibit neural activities in the brain. Recent research shows the possibility of using ultrasound ranging from 0.5 to 43 MHz in acoustic frequency to activate the retinal neurons without causing detectable damages to the cells. This review recapitulates pilot studies that explored retinal responses to the ultrasound exposure, discusses the advantages and limitations of the ultrasonic stimulation, and offers an overview of engineering perspectives in developing an acoustic retinal prosthesis. For comparison, this article also presents studies in the ultrasonic stimulation of the visual cortex. Despite that, the summarized research is still in an early stage; ultrasonic retinal stimulation appears to be a viable technology that exhibits enormous therapeutic potential for non-invasive vision restoration.

## 1. Introduction

Outer retinal degenerative diseases are common causes of blindness, characterized by the progressive deterioration of the photoreceptors that results in permanent vision loss [1,2]. Two of the most prevalent forms of such diseases are Age-related Macular Degeneration (AMD) and retinitis pigmentosa (RP). AMD is a leading cause of blindness in the elderly population, estimated to affect 196 million people globally by 2020 [3,4,5]. Dry AMD, also known as geographic atrophy (GA) or atrophic AMD, accounts for 85–90% of all AMD cases [6]. In dry AMD, retinal atrophy arises from accumulation of yellow deposits called drusen in the macula between the retinal pigment epithelium (RPE) and choroid. Retinitis pigmentosa (RP) is the most common form of inherited retinal degeneration, with a worldwide prevalence of 1/3000 to 1/4000 people [7,8,9,10]. In RP, initial impairment in the peripheral vision and night vision is typically followed by progressive loss of central vision, leading to complete blindness.

**Figure 1:**
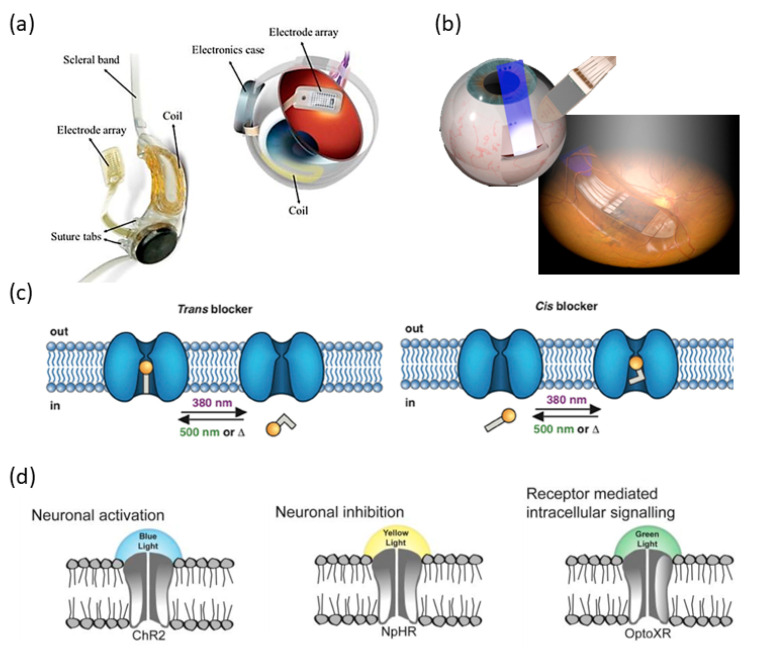
Illustration of the vision restoration approaches in the retina with (**a**) an epiretinal prosthesis (image from Bloch et al., 2019, [13]), (**b**) a subretinal prosthesis (image from Edwards et al., 2018 [15]), (**c**) chemical photoswitches (image from Mourot et al., 2013 [21]), and (**d**) optogenetic tools (image from Pama et al., 2013 [23]).

Currently, there is no approved medical intervention that could cure or reverse the courses of dry AMD and RP. In both conditions, a significant number of inner retinal neurons downstream of the photoreceptor layer remain capable of functioning, despite significant remodeling and reorganization of the retinal circuitry (refer to Masland et al. [11] and Marc et al. [12] for a review). These surviving neurons can be directly stimulated, bypassing the damaged photoreceptors. Inner retinal stimulation has drawn significant attention in the past two decades, spawning a number of studies in two major vision restoration approaches: (1) Bioelectronic and optoelectronic retinal implants and (2) inner retinal modifications with optogenetic tools and photochemical switches (Figure 1).

The former approach typically involves encoding visual inputs into artificially generated and amplified electrical currents. This strategy has resulted in several clinically implemented retinal implants: Argus II epiretinal prosthesis (Second Sight Medical Products, Sylmar, CA, USA), Alpha IMS/AMS subretinal prosthesis (Retina Implant AG, Reutlingen, Germany), and PRIMA subretinal prosthesis (Pixium Vision, Paris, France). Argus II and Alpha IMS/AMS are clinically approved for RP treatment while PRIMA is being tested as a plausible treatment for AMD [13,14,15,16]. In 2019 and 2020, IMS/AMS and Argus II were successively discontinued. Advances in organic electronics and nanotechnology have produced new materials that are being actively investigated as alternative photocurrent sources, potentially providing improved visual acuity and reduced system complexity [17,18].

The latter approach typically involves rendering light responsiveness to the remaining retinal neurons that are not intrinsically sensitive to light, by membrane modification with the expression of optogenetic proteins (e.g., channelrhodopsin [19,20] and halorhodopsin [1]) or with the attachment of photoswitchable ligands [21]. With the design of proper light stimulus delivery methods, e.g., the holographic stimulation pattern proposed by Reutsky et al. [22], this strategy could potentially achieve a spatial resolution of single-cell precision. Yet challenges in light sensitivity, expression uniformity, molecule stability, response rate, and biosafety need to be addressed for these approaches to be clinically employed.

This review article focuses on yet another inner retinal strategy that is being explored for vision restoration, leveraging the more recently discovered ultrasonic activation of the retina. Development of this strategy is still in an early stage. Here we will discuss some of the pilot studies in the physiological mechanisms of the retinal responses to ultrasound, advantages and limitations of the ultrasonic stimulation, as well as some engineering aspects of the design of an acoustic retinal prosthesis for clinical application.

## 2. Ultrasonic Retinal Stimulation

### 2.1. Ultrasonic Neuromodulation

Ultrasound is an acoustic wave of frequencies higher than the upper limit of sound perception of human ears (20 kHz). Ultrasound waves may be generated by transducers made from piezoceramic materials that convert electrical energy into mechanical vibration. In recent years, ultrasonic neuromodulation has gained traction for its minimal invasiveness, spatial precision of stimulation, and compatibility with neuroimaging devices [24].

Ultrasonic stimulation of the neurological tissues was pioneered by Edmund Harvey in 1929 in ex vivo frog and turtle ventricular muscles [25]. He reported seeing muscle contractions upon the delivery of high intensity continuous ultrasonic stimulation. Later, more direct evidence of the ultrasonic effects on the nervous system was revealed, demonstrating that neural activity could be both excited and inhibited by the ultrasound. In 1958, Fry et al. showed in vivo the reversible suppression of the sensory-evoked potentials in the central nervous system by the ultrasound [26]. Exposure of the cat lateral geniculate nucleus (LGN) to focused ultrasound (980 kHz) through a cranial window produced reversible suppression of the light-evoked potentials by over 67%, and the complete recovery took about 30 min [26,27,28,29]. In 1976, Gavrilov et al. demonstrated in human that the focused ultrasonic stimulation ranging from 0.48 to 2.67 MHz excited a variety of superficial and deep peripheral nerves, such as those mediating thermal, pain, and tactile sensations [30]. Tufail et al. showed in mice that low-frequency ultrasound pulses with 36.2 mW/cm^2^ I_SPTA_ (Spatial Peak, Time Average Intensity) at 0.3 MHz or with 64.53 mW/cm^2^ I_SPTA_ at 0.5 MHz were effective in modulating mammalian cortical and hippocampal activities in vivo without damaging the brain [31]. Lee and colleagues found that sonication of the primary somatosensory cortex elicited transient tactile sensations on the hand area contralateral to the sonicated hemisphere, with anatomical specificity of up to a finger. Collaborating the behavioral results, electroencephalography (EEG) recordings revealed sonication-specific evoked potentials. In a later study, Lee et al. sonicated the primary visual cortex (V1) and monitored brain activity with functional magnetic resonance imaging (fMRI). They found activation elicited not only in the sonicated brain area, but also from the network of regions involved in visual and higher-order cognitive processes. These and other pioneering studies revealed the possibility of using ultrasound as a non-invasive tool to modulate a wide range of neural activity in the central and peripheral nervous systems [32,33,34,35,36].

### 2.2. Ultrasonic Retinal Stimulation

Focused ultrasonic stimulation of the retina was first reported by Naor et al., who collected visually evoked potentials (VEPs) under the retinal stimulation with low-frequency ultrasound (0.5 MHz and 1 MHz) in the anesthetized wild-type Sprague–Dawley rats [37]. This in vivo study did not look specifically at the neural response on the retinal level, but the investigators envisioned an ultrasound retinal prosthesis that is capable of simultaneous multifocal stimulation at a large visual angle. The prosthesis consists of an external camera with an image processor and an ultrasound phased array interfaced with the cornea via an acoustic coupling component to project complex acoustic images onto the retina.

Later, Menz et al. characterized responses of the isolated salamander retinas to high-frequency ultrasonic stimulation. Stimuli at an acoustic frequency of 43 MHz were delivered focally with a spot diameter of 90 μm, evoking both ON and OFF responses with a temporal precision similar to the visual responses but with a shorter latency. Pharmacological blockage of the synaptic transmission suggested that ultrasonic stimulation did not directly activate retinal ganglion cells, but the interneurons beyond photoreceptors. Such findings may imply a selective effect of the ultrasound on ion channels or other membrane proteins.

More recently, Jiang et al. evaluated responses of the rat retinal ganglion cells in the wholemount configuration to the ultrasonic stimulation of a lower frequency (2.25 MHz) [38]. Comparison of the multielectrode array (MEA) recording from the ganglion cell layer under ultrasonic vs. light stimulation showed differential ON/OFF dynamics and temporal properties for the two stimuli, including latency and firing rate. A particularly interesting observation associated with the ultrasonic stimulation is the dual-peak pattern of the response—an early transient burst followed by a later sustained response upon the onset or offset of the stimulus; additionally, the early transient phase diminished faster than the sustained phase with the decrease of the ultrasound intensity. Due to the limited spatial resolution of the MEA recording performed, each recorded trace may contain a mix of spiking activity from multiple ganglion cells nearby the recording electrode. As such, it remained unclear if the two response peaks originated from the same cell or two neighboring cells. Single-cell recording is perhaps needed to better understand the neural mechanism of the ultrasonic stimulation in the retina. Nonetheless, on the population scale, the dual-peak pattern may have implications on the visual sensations that could be produced by the ultrasound, as temporal characteristics of the ganglion cell responses encode important aspects of visual information [39,40,41,42].

### 2.3. Possible Mechanisms of Ultrasonic Neuromodulation

#### 2.3.1. Thermal Effects

Ultrasound can produce both thermal and nonthermal effects in biological tissues. Temperature rise in the tissue due to absorption of the ultrasonic energy is dependent on factors such as ultrasound parameters (e.g., acoustic frequency, pulse rate, intensity, and exposure time) as well as tissue characteristics (e.g., acoustic impedance) [43,44].

Over the past decade, impact of temperature elevation on neural activation has been studied in the optical stimulation [45,46,47]. Infrared light was found to cause a rapid increase in the temperature of the membrane, leading to neural excitation [45,48,49]. Two primary mechanisms of the opto-thermally driven neural excitation were hypothesized: (1) Change in the capacitance of the plasma membrane with temperature [45,48,49] and (2) temperature-triggered activation of the thermosensitive ion channels [50,51].

For the ultrasound stimulation, thermal mechanism has been considered less of a concern in the past as the temperature increase during ultrasound exposure was shown to be minimal (less than 0.1 °C in most studies) [31,32,52,53,54]. Tufail et al. monitored the temperature change of the motor cortex under different pulse durations with a thermocouple inserted through the cranial window. No significant cortical temperature change was observed with the pulse duration ≤0.57 ms and the peak negative pressure <0.097 MPa. At the peak negative pressure of 0.1 MPa, pulse duration >50 ms produced merely a 0.02 °C rise in the temperature [31]. Yoo et al. found no measurable temperature change by the non-invasive magnetic-resonance thermometry with the focal stimulation (1-s duration and 12.6 W/cm^2^ I_SPPA_ (Spatial Peak Pulse Average Intensity)) that produced a visible forepaw movement in the rabbits [52].

However, the above-summarized ultrasound studies all presented macroscopic temperature changes, which are distinct from the microscopic temperature changes measured in the opto-thermal studies by pipet resistance [45,55] or fluorescent microthermal imaging [46]. Hence, it awaits to be determined if ultrasonic stimulation causes a substantial microscopic temperature change in the target tissues. If such an acousto-thermal effect exists, according to the finding by Farah et al. [46], membrane current may be determined by the change rate of the temperature. Interestingly, a recent study suggested that altered membrane capacitance could underlie the ultrasonic neural activation [56], echoing with one of the two primary hypotheses of opto-thermal stimulation.

#### 2.3.2. Nonthermal Effects

Nonthermal mechanisms of the ultrasonic neuromodulation are not yet fully understood. Possible roles of the mechanical effects of ultrasound have been probed in terms of radiation force, microbubble cavitation [24,57], intramembrane cavitation between the bilayer membrane leaflets [58], and the mechanosensitive ion channels.

Radiation force is a continuous, non-oscillating force created by an acoustic wave propagating in an attenuating medium. The momentum transfer from the wave to the medium, via scattering or absorption, produces a gradient of acoustic energy density. Radiation force of a plane wave in an absorbing medium can be presented by the following equation [59,60,61,62]:

(1) $$F=\frac{2\alpha I}{c}$$

where *α* is the attenuation coefficient of the medium, *I* is the local temporal average intensity, and *c* is the speed of sound traveling in the medium. By this mechanism, a high-frequency acoustic wave can be converted into a low-frequency mechanical force with dynamics of the envelope of the wave.

Cavitation is the interaction between gas bubbles and the sound field, including inertial cavitation and non-inertial cavitation. Inertial cavitation (also termed transient cavitation) occurs under suprathreshold acoustic pressure that leads to rapid expansion followed by violent implosion of the bubbles. In non-inertial cavitation, formerly termed stable cavitation, bubbles undergo repetitive oscillation over multiple cycles and the size change of the bubbles typically does not exceed twice the equilibrium radius [63,64,65]. Cavitation decreases at higher frequencies due to the difficulty to sustain oscillation in the bubble.

An alternative neuronal intramembrane cavitation hypothesis, ‘bilayer sonophore’ (BLS) model, was proposed by Krasovitski et al. at 2011 [58]. In this model, the oscillating acoustic pressure forms gas bubbles in the intramembrane space between the two leaflets of the lipid membrane. The leaflets are pulled away in response to the negative pressure and are pushed together under the positive pressure, which causes a change in the membrane permeability, activating mechano-sensitive proteins [66]. The mechanosensitive ion channels are transmembrane proteins that respond to mechanical stress by conformational change. It has been reported that the focused ultrasound elicited transmembrane current flow through mechanosensitive sodium, calcium, and potassium channels [32,67,68,69]. It is also possible that these channels are directly activated by the radiation force, without the need of membrane cavitation.

Given these possible mechanisms, a recent study by Menz et al. set out to understand the main driving force of the ultrasound sensitivity in the retina. By means of two-photon laser-scanning microscopy, they measured the microscale tissue displacement in the ex vivo salamander retina during the ultrasound exposure at the same time when the firing activity of the ganglion cells was recorded. Under variations of acoustic intensity, they found a positive correlation between the displacement and the normalized firing rate, consistent with the nonlinear effect of the radiation force. Furthermore, they varied the acoustic frequency in a wide range between 0.5 and 43 MHz, finding enhanced retinal activity under higher frequencies. This observation argues against any major direct role played by cavitation in ultrasonic stimulation of the retina, as cavitation tends to diminish when the frequency is increased [70].

## 3. Spatiotemporal Characteristics of Retinal Response to Ultrasonic vs. Visual Stimulation

### 3.1. Temporal Characteristics

**Figure 2:**
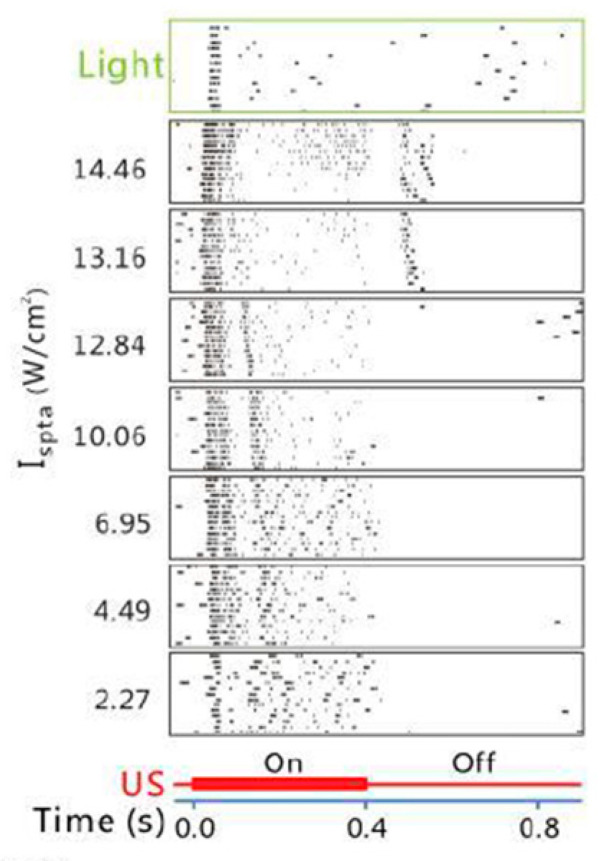
Shift in the response pattern of the retinal ganglion cells with increase of the acoustic intensity. The ON-transient cells (classified by the light response) responded to the onset of the ultrasonic stimuli at lower intensity but responded to both the onset and offset of the ultrasonic stimuli when the ultrasound intensity was increased. (Image from Jiang et al., 2018 [38].)

Temporal profiles of the ganglion cell spiking activity under ultrasonic vs. visual stimulation were compared [54]. Some cells exhibited similar firing rate and response duration for the two stimuli but shorter response latency for the ultrasonic stimulation. The shorter latency was attributed to the activation of the retinal neurons downstream of photoreceptors, bypassing the phototransduction cascade. Some other cells responded to both onset and offset of the ultrasound stimuli but only responded to the offset of the visual stimuli. Similar observations in the ultrasonic vs. visual stimulation were noted by Jiang and colleagues [38]. By classifying the ganglion cells into four types (ON-transient, ON-sustained, OFF-transient, and ON/OFF cells) based on their light responses, the investigators found that the ON and OFF patterns of the ultrasonic response did not align with the light response. For example, the ON-transient cells responded to the onset of the ultrasonic stimuli at the low ultrasound intensity, and they responded to both the onset and offset of the ultrasonic stimuli when the ultrasound intensity was increased (Figure 2). It is not clear whether such differences arose from the same cells responding differently to the two stimuli or different cell populations being activated. Correlation between the acoustic intensity and the temporal characteristics of the response may imply engagement of a mix of ON and OFF cells in responding to enhanced acoustic power that contributed to a shift from the single-sided ON or OFF response to the double-sided ON and OFF response.

Jiang et al. also noted a dual-peak pattern in the ultrasonic response of the ganglion cells, described in Section 2.2. A similar dual-peak pattern to light stimuli was previously found in several different species, including chicken, turtle, frog, and mouse [71,72]. It has been hypothesized that the two peaks were generated through different neural pathways—the first peak generated by the signal transmission from photoreceptors to bipolar cells and on to ganglion cells, whereas the second peak arose from the lateral inhibition of the retinal circuitry [72,73]. It awaits to be determined if the second peak manifested under ultrasonic stimulation shares similar mechanistic origins with that observed in the light stimulation, as well as its dependence on the acoustic frequency and the possible role in shaping the visual perception.

By overlapping the ultrasound stimuli with the visual stimuli, Menz et al. measured how the ultrasound modulated the natural visual processing of the retina. They found that, although the ultrasound did not fundamentally change the temporal filtering, it modulated visual sensitivity and threshold in different manners for different cells [54]. It will be interesting to find out in the future how such modulation could impact the integration of the ultrasonic vision with the natural one, for example, in AMD patients with some remnant vision in the periphery of their visual field.

### 3.2. Spatial Characteristics

In normally sighted people, photoreceptors can differentiate slight changes in the spatial patterns of the incident light, owing to the small receptive field of a single photoreceptor. The cross-section of the outer segment of rod and cone photoreceptors ranges between 0.5 to 4 µm, comprising the basic light-sensing element for optical stimulation. Correspondingly, the 20/20 vision represents the ability to resolve two points separated by 1 arcmin, equivalent to 4.5 µm on the retina. In the current bioelectronic retinal prostheses that are designed to stimulate bipolar and/or ganglion cells with electrical currents, due to the current dispersion, visual acuity is determined not only by the spacing of electrodes but also by the electrical contact with the target neurons as well as their receptive field (see Eiber et al. [74] for a review). The best acuity clinically restored by bioelectronic visual prosthesis so far is 20/400 in patients visually impaired by advanced macular degeneration [14].

Spatial resolution of the ultrasonic stimulation is mainly dependent on the acoustic frequency and aperture size. In general, a higher acoustic frequency (shorter wavelength) will lead to a better axial resolution but worsened attenuation as it is more easily absorbed by the ocular tissues along the acoustic path [75]. Likewise, lower acoustic frequency will result in a lower spatial resolution but less attenuation, allowing deeper penetration into the ocular structures. Unlike brain stimulation, retinal stimulation does not require penetration through the bones; therefore it is feasible to use higher frequencies. Acoustic attenuation in the eyes is less than 0.5 dB at 1 MHz and ~1% of the incident energy is reflected [76,77,78]. Naor et al. [20] suggested that acoustic retinal stimulation in the 2–10 MHz range could achieve a spatial resolution similar to what is provided by Argus II, the FDA-approved retinal prosthetic device.

Spatial resolution of acoustic stimulation is typically quantified by the mean full width at half maximum (FWHM) of the focal plane. The longitudinal and transverse acoustic intensity profiles of the acoustic focus are examined by scanning the area around the focal region using the hydrophone. To our knowledge, the highest resolution reported for laboratory investigation of ultrasonic retinal stimulation is ~100 µm with the 43 MHz stimuli [54]. It may be possible to achieve a resolution of 30 μm with a 70 MHz transducer [79,80] or even a resolution of on the scale of 10 μm with a higher-frequency transducer [81]. However, previous studies indicated a reduced stimulation efficiency with the increase of the frequency. Therefore, perhaps a trade-off between efficiency and spatial resolution needs to be taken into consideration [54,82]. The current discussion is limited to the theoretical estimation with respect to the frequency, but the actual beam width of the ultrasound is also determined by the transducer parameters such as focal length and aperture, as shown by the Equation (2). In future studies, the spatial acuity that is needed to achieve significant gain of function for the blind can be evaluated by psychophysical approaches, similar to those adopted by the bioelectronic retinal prostheses [16].

(2) $$\mathrm{Width}\;\mathrm{of}\;\mathrm{focused}\;\mathrm{beam}\approx \frac{\mathrm{focal}\;\mathrm{length}\times \mathrm{wavelength}}{\mathrm{aperture}}$$

Retinal ganglion cells have an antagonistic center-surround receptive field, rendering an ON center with an OFF surround or an OFF center with an ON surround. This spatial opponency plays a fundamental part in many aspects of the visual processing, from contrast detection to color sensation [83,84,85]. To determine whether the spatial antagonism is preserved in ultrasonic stimulation, Menz et al. measured the response of the salamander retinas to focused ultrasound stimulation as a function of lateral distance from the ganglion cells [54]. They found that moving the transducer away from the receptive field center led to the cells transitioning from responding to the offset of the ultrasound stimuli to responding to the onset of the stimuli. Similar to the receptive field of visual stimuli, the antagonistic surround spanned a larger region than the center. More research is needed to determine if this center-surround organization persists in the face of retinal remodeling during degeneration. Nonetheless, this finding implies the possibility of leveraging spatial antagonism in ultrasonic stimulation of the retina. Focused stimulation with higher acoustic frequencies may thus enable single-cell activation, achieving patterned stimulation with high spatiotemporal precision.

## 4. Acoustic Retinal Prosthesis (ARP)

### 4.1. Basic Architecture and the Transducer Array

**Figure 3:**
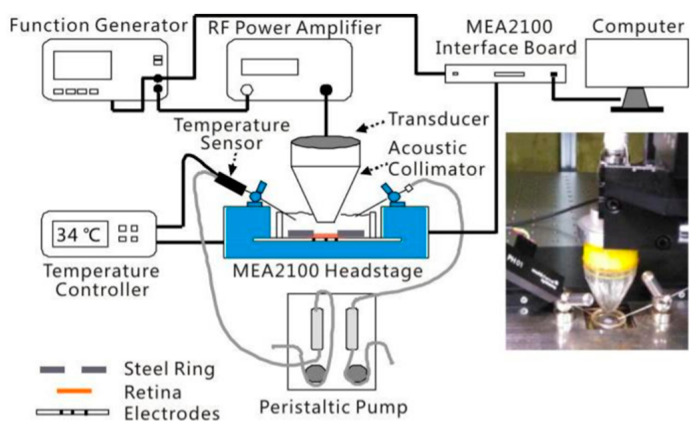
An experimental setup for in vitro retinal stimulation with a single-element transducer. (Image from Jiang et al., 2018 [38].)

An ARP needs to efficiently and accurately generate acoustic patterns on the curved surface of the retina. Such a device may consist of (1) an image acquisition unit to capture the visual scenes, (2) an image processing unit to convert visual scenes into ultrasonic stimulation patterns, and (3) a transducer array that generates patterned stimulation on the retina. Single-element focused ultrasound transducers have been used for single-site ultrasound neurostimulation in the brain [31,32,33,52,54,82,86,87] and in retina in vitro [38] (Figure 3), but it is not feasible for an ARP that requires multifocal stimulation to generate useful form vision. Hence, a multi-element array transducer will be employed [57,88,89].

**Figure 4:**
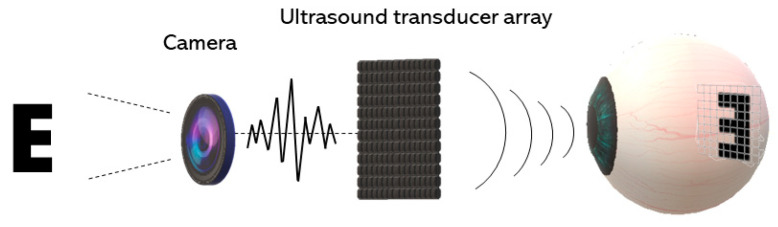
Scheme of an acoustic retinal prosthesis (ARP). The visual scene captured by an external camera is processed and transmitted to an ultrasound transducer array for stimulation in the retina.

A scheme for an ARP is illustrated in Figure 4, similar in concept to what was first proposed by Naor et al. [37]. The multi-element array can be placed non-invasively on the cornea and, if needed, coupling gel will be applied between the array and the cornea to minimize the reflection of the acoustic waves from the boundaries. The acoustic waves will penetrate through the eye from the cornea to the retina and create a projected pattern in the retina [90].

Gao et al. proposed a contact lens array transducer that utilizes the tear film for acoustic coupling between the transducer and the cornea [91]. The array is flexible and can be placed outside the eyeball, resembling a contact lens. The overall array is 8.7 mm in radius with 256 ultrasonic elements covering the pupil. The acoustic wave will have a focal depth of 24 mm, approximating the diameter of an adult eyeball [92]. At 2.5 MHz acoustic frequency, the lateral resolution is estimated to be 1.3 mm.

**Figure 5:**
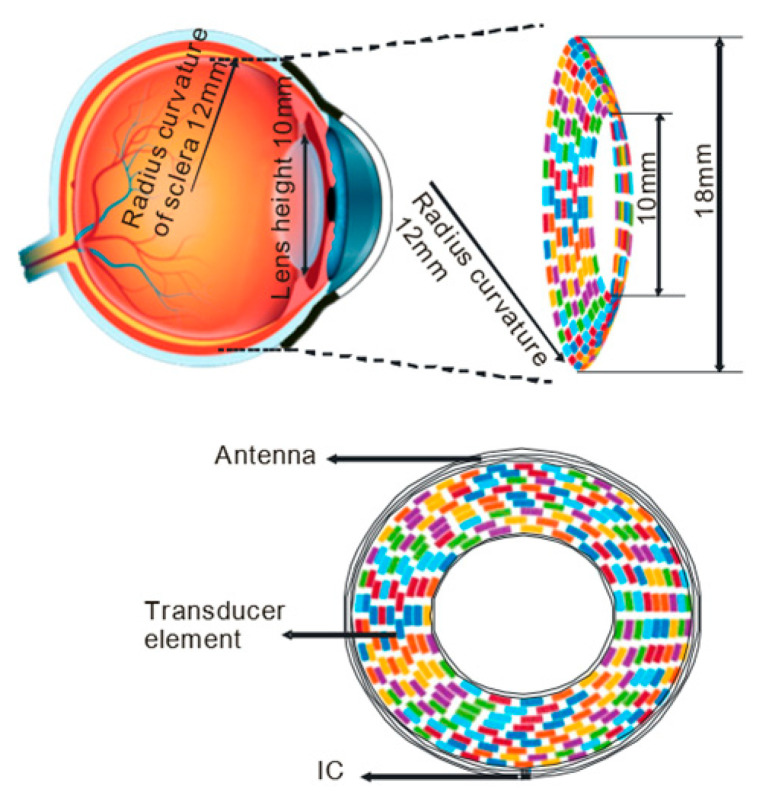
Design of a racing ring lens ultrasound transducer array for multifocal retinal stimulation. (Image from Yu et al., 2019 [93]).

A new 5MHz racing (circular) array transducer was recently proposed by Yu et al. [93], which also mimics a contact lens but with an opening in the center to minimize the exposure of the intraocular lens tissue to the ultrasound (Figure 5). Lens has a high acoustic absorption (7.8 dB/cm @10 MHz) [75], especially when high-frequency ultrasound is used. The excessive acoustic absorption may cause unwanted, potentially damaging, heating effect. This open-ring design minimizes direct ultrasound exposure in the lens, making it easier to increase the acoustic frequency to improve the stimulation resolution. A simulation of this design shows that the resolution could be improved from 1.3 mm to 0.6 mm by increasing the acoustic frequency from 2.5 MHz to 5 MHz [91,93].

Side lobes and grating lobes are the secondary lobes of ultrasound beams in the directions different from that of the primary beam. For a transducer array, grating lobes may occur when the spacing of the elements is equal to or greater than half the wavelength. These secondary lobes may lead to distorted activation patterns, thus should be suppressed to a satisfactory level. To diminish the grating lobe-caused artifact, pitch size should be at the wavelength level, which means that higher frequencies will demand smaller elements and, thus, more challenging fabrication techniques.

In totally blind patients perhaps the most important aspect of vision restoration is to gain independent mobility, which requires broader peripheral stimulation. This is ideal for the contact lens design which can be easily configured to stimulate broadly, whereas to cover a similar stimulation area, the electrode array in a bioelectronic retinal prosthesis will be too large to conveniently implant.

### 4.2. Algorithms for Generating Multifocal Ultrasonic Stimulation

Restoring basic spatial visual functions requires the ability of an ARP to mimic the parallel visual inputs by multifocal stimulation. Thus, algorithms previously developed to optimize the ultrasound distribution under the context of hyperthermia research may find applications in retinal stimulation [57,94,95]. The pressure field generated by focused ultrasound transducers can be calculated by Rayleigh–Sommerfeld or Fourier transform. Both algorithms are discrete in that each transducer element is considered a source point and these sources generate superimposed ultrasound patterns at each point of the target plane.

The Rayleigh–Sommerfeld [57] transform creates complex ultrasound patterns at different positions. The pressure at point r is given by:

(3) $$p\left(r\right)=\frac{j\rho ck}{2\pi }\overset{}{\underset{S}{\int }}u\left(r^{\prime }\right)\frac{e^{-jk\left(\left|r-r^{\prime }\right|\right)}}{\left|r-r^{\prime }\right|}dS$$

where *p*(*r*) is the acoustic pressure at the observation point, *c* is the velocity of sound traveling in the medium, ρ is the density of the medium, k is the wavenumber of the ultrasound, *S* indicates the surface of the source area, and *u* is the excitation of the source point on the transducer at point *r*′.

The equation can be discretized (N elements of the transducer and M points on the target plane) and written in the matrix form:

(4) $$p=H\hat{u}$$

where *p* is the pressure amplitude at the target plane (M × 1), $\hat{u}$ is the excitation vector of the array elements (N × 1), and *H* is the propagation operator (M × N).

Several algorithms were developed to solve the excitation field from the Rayleigh–Sommerfeld equation. A “pseudoinverse” method was proposed to invert the propagation operator of the minimum norm solution [57]:

(5) $$\hat{u}={\left(H^{*}\right)}^{t}{\left(H{\left(H^{*}\right)}^{t}\right)}^{-1}p$$

where ${\left(H^{*}\right)}^{t}$ is the conjugate transpose (the adjoint) of *H* and ${\left(H^{*}\right)}^{t}{\left(H{\left(H^{*}\right)}^{t}\right)}^{-1}$ is the pseudoinverse of *H* that can be evaluated by the spectral value decomposition (SVD). This method produces precise pressure intensity with good uniformity. Additionally, the excitation efficiency can be improved by introducing the weighting matrix into iteration. The “conjugate field method” dropped the pre-emphasis term, ${\left(H{\left(H^{*}\right)}^{t}\right)}^{-1}$, and, thus, is perhaps less precise compared to the pseudoinverse method [95].

Gerchberg–Saxton (GS) algorithm is an iterative optimization algorithm developed to retrieve the phase distribution of a propagating function, if the irradiance information on those planes is known [96]. The algorithm was extended to weighted Gerchberg–Saxton (GSW) by Hertzberg et al. in 2010 to increase the efficiency and focal spot uniformity [97]. For a 3D patterned stimulation, the GSW algorithm can be potentially applied to increase the amplitude uniformity on a curved surface [91].

Wu et al. simply performed the non-iterative fast Fourier transform to calculate the transducer function. For the far field (Fraunhofer region), the pressure pattern *P*(*x*,*y*,*z*) on an x-y plane from the transducer *S*($x_{0},y_{0},0$) can be defined as [98,99]:

(6) $$P\left(x,y,z\right)=\frac{1}{z}e^{\frac{jk\left(x^{2}+y^{2}\right)}{2z}}\int_{-\infty }^{\infty }\int_{-\infty }^{\infty }S\left(x_{0},y_{0}\right)e^{-jk\left(\frac{x_{0}x+y_{0}y}{z}\right)}dx_{0}dy_{0}$$

where λ is the wavelength and *k* is wavenumber equals 2π/λ. The 2D Fourier transform of the source with *u* = *x*/*λz* and *v* = *y*/*λz* is:

(7) $$P\left(x,y,z\right)=\frac{1}{z}e^{\frac{jk\left(x^{2}+y^{2}\right)}{2z}}F\left\{S\left(x_{0},y_{0}\right)\right\}.$$

The transducer function can be solved by the inverse Fourier transform, the reduced computational complexity of which can facilitate real-time implementation. However, the derivation of this algorithm is for far field only and it does not allow excitation of all elements at full power (maximum amplitude) as it is both amplitude- and phase-modulated [100].

### 4.3. Ultrasonic Stimulation Strategy

Choice of the acoustic frequency determines the spatial resolution and greatly impacts the stimulation efficiency, as discussed in Section 3.2. Intensity of the ultrasound was found to modulate firing rate, latency, and even response patterns of the ganglion cells. The power threshold for the ultrasound waves to induce in vivo neuromodulation was found to be about 0.25 W/cm^2^ [82]). In general, an increase in the ultrasound intensity leads to an increased firing rate and a decreased latency until the response reaches saturation [54]. In addition, variations in the acoustic intensity may also shift response patterns of a given ganglion cell or between different cell populations, as discussed in Section 3.1 [38]. Menz et al. found that the pulse repetition rate and pulse duration had no effect on the responses when the average power was held constant, highlighting the importance of the average power [54].

**Figure 6:**
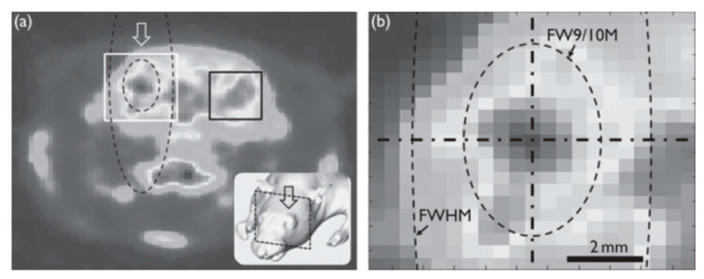
(**a**) Sonication effect illustrated by a PET image. The arrow indicates the sonication path and the black rectangle marks an unsonicated region. (**b**) A close-up view of the acoustic focus outlined by the white square in (**a**). The dashed lines encircle the full width at 90% maximum (FW9/10M) area (inner) and the full width at half maximum (FWHM) area (outer), respectively. (Image from Kim et al., 2014 [104]).

Cigar-shaped focal volume of the ultrasound field with an aspect ratio between 0.16~0.57 has been described in ultrasonic neuromodulation [52,86,101,102,103,104,105,106,107,108]. The cigar-shaped sonication volume was suggested to cause multi-layer neural stimulation, which, depending on the application, could facilitate a multi-depth stimulation but with a sacrifice of the spatial specificity [91,103]. Kim et al. (2014) contended otherwise. They used standardized uptake value (SUV) to measure the sonication effect at different brain depths—the SUV is higher in the modulated regions than in the unsonicated area. Both the full width at 90% maximum (denoted as ‘FW9/10M’) and the full width at half maximum (FWHM) of the acoustic field exhibited a cigar-shaped contour (Figure 6a), covering a significant depth range, but the actual modulated region was much more focused with a rounder contour (Figure 6b, darker area) [104]. Hence, this study suggests that the activated region does not necessarily reflect the spatial profile of the acoustic power distribution. More mechanistic investigation is required to understand why.

It should be noted that in a design that places the transducer array outside the eye, different mechanical properties of different eye tissues along ultrasound propagation path and localization of the mechanosensitive receptors in these tissues (e.g., cornea as a high density of mechanosensitive nocioceptors) could potentially impact efficacy and safety of the retinal stimulation, and, thus, need to be taken into consideration in the optimization of the transducer array.

### 4.4. Safety Consideration

Biological safety of the ultrasound depends largely on the power intensity. Lower intensities produce reversible neuromodulation, whereas higher intensities could lead to cell death [109,110]. Ultrasound intensity, therefore, should be calibrated with caution, particularly for the chronic use. The guidelines for ophthalmology applications of the ultrasound set by the US Food and Drug Administration (FDA) are given in Table 1 [111]. These guidelines are more restrictive than those for other applications, perhaps partly due to the excessive absorption of the acoustic energy by the lens. Currently there is no regulation established specifically for the ultrasonic neurostimulation, so the ultrasound intensity delivered by an ARP should at least conform to the standard set for the general ophthalmological purposes.

## 5. Ultrasonic Stimulation of the Visual Cortex

Vision restoration with the ultrasound is not limited to a retina-based mechanism, given the accumulating evidence of the susceptibility of the visual cortex and other higher visual centers to ultrasonic stimulation. Focused ultrasound delivered to the cats’ lateral geniculate nucleus was demonstrated to reversibly suppress the VEPs [26]. Ultrasound transcranially delivered to the primary visual cortex (V1) in sheep, whose skull has a thickness similar to that of human, elicited electroencephalographic potentials [35,112]. In human subjects, single-element focused ultrasound stimulation of the V1 elicited phosphene sensation (mostly colorless, patternless, and shapeless). Brain activation map obtained with fMRI showed that sonication in this area was also associated with the higher-order visual and cognitive processing and no adverse effects were found by the neurological examinations [36]. These results encourage exploration of acoustic cortical stimulation as a potential approach to restoring sight, perhaps in patients blinded by neuropathology further along the visual pathway with respect to the retina. Yet, transcranially delivered ultrasound needs to pass through the skull to reach the target area, potentially limiting the spatial resolution of activation. See Table 2 for a list of representative literatures on the ultrasonic retinal and visual cortical stimulation.

## 6. Conclusions

In this paper, we reviewed pioneering studies in ultrasonic stimulation of the retina. Ultrasound presents an emerging method for non-invasive neuromodulation. Though current studies demonstrate the potential to acoustically stimulate and modulate retinal activity without causing unwanted tissue damage, more work is required to further identify the underlying neural mechanisms and reveal the optimal strategies to efficiently activate the retina with high spatiotemporal precision. Advanced understanding in these areas may one day lead acoustic retinal prosthesis from conceptualization to a clinically implementable device.

## Figures and Tables

**Table 1:** Safety guidelines for ophthalmological ultrasound applications, according to [111].

Index	Definition	Safety Limit
Spatial peak time average intensity (I_SPTA_)	The maximum intensity measured within the sound field averaged over the sonication time.	I_SPTA_ ≤ 50 mW/cm^2^
Spatial peak pulse average intensity (I_SPPA_)	The maximum intensity measured within the sound field averaged over the duration of a single pulse	I_SPPA_ ≤ 50 mW/cm^2^
Mechanical index (MI)	$\mathrm{MI}=\frac{\mathrm{peak}\;\mathrm{negative}\;\mathrm{pressure}}{\sqrt{\mathrm{center}\;\mathrm{frequency}\;\mathrm{of}\;\mathrm{the}\;\mathrm{US}\;\mathrm{beam}}}$	MI ≤ 0.23
Thermal index (TI)	$\mathrm{TI}=\frac{\mathrm{Transcuder}\;\mathrm{power}\;\mathrm{exposing}\;\mathrm{the}\;\mathrm{tissue}}{\mathrm{The}\;\mathrm{power}\;\mathrm{requred}\;\mathrm{for}\;\Delta T=1\;{}^{{}^{\circ}}C}$	TI ≤ 1

**Table 2:** Summary of the representative literatures on ultrasonic retinal and visual cortical stimulation. (Information compiled from a literature search for published studies as of June 2020 on retinal neuromodulation and transcranial visual cortical stimulation by ultrasound.)

Authors	Transducer	Acoustic Frequency (MHz)	Resolution * (mm)	I_SPPA_ (W/cm^2^)	PRF (Hz)	Duty Cycle (%)	Stimulation Time (s)	Region	Species	Major Findings and Experimental Outcomes
Yoo et al. [52]	single element	0.69	2.3	3.3–12.6	10–1000	5	0.5–2, 9	V1, M1	Rabbit (in vivo)	Ultrasound-induced excitation and inhibition of the neural activity.
Lee et al. [112]	single element	0.25	47	1.7–14.3	500	50	0.3	V1	Sheep (in vivo)	Highly variable threshold acoustic intensity for focused ultrasonic stimulation. Possibility of hemorrhage.
Lee et al. [36]	single element	0.27	3	16.6	500	50	0.3	V1	Human (in vivo)	Demonstrated ultrasound modulated activities in the primary somatosensory cortex and ultrasound induced phosphene perception.
Kim et al. [113]	single element	0.35	3.7 (The full-width at 90% maximum)	1, 3, 5	100	1, 5, 8.3	150	Visual cortex	Rat (in vivo)	VEP was evoked or suppressed depending on the intensity and duty cycle of the acoustic wave
Naor et al. [37]	phased array	0.5, 1	0.4–0.53	0.1–0.4, 5.2–8.5	1900–2000, 1667	10~20	5~20	RGCs	Rat (in vivo)	Conceptualized an acoustic retinal prosthesis and adapted the algorithms to generate spatially patterned multifocal stimulation.
Menz et al. [54]	single element	43	~0.1	20~60	0.5–1 M	100	1	RGCs	Tiger salamander (in vitro)	Conducted high frequency retinal stimulation and demonstrated a spatial precision of ~100 um.
Jiang et al. [38]	single element	2	1.6	12.84 (I_SPTA_)	1000	50	0.4	RGCs	Rat (in vitro)	Found the difference in the response pattern of the RGCs to light vs. ultrasound stimuli, and the dual-peak responses to ultrasound that are intensity dependent.
Gao et al. [91]	contact lens	6~0.3	12.5–5	8.1, 9.3, 10	1000	-	0.3	RGCs	Simulation	Proposed a contact lens form transducer array that utilizes the tear film for acoustic coupling.
Yu et al. [93]	racing array	2.5, 5, 10	1.3, 0.6, 0.26	0.2–0.6	-	-	-	RGCs	Simulation	Proposed a racing ring lens design to avoid the acoustic exposure of the lens suitable for high frequency stimulation.
Lu et al. [107]	single element	0.5	2.4 (The full-width at 25% maximum)	115.8	100–500	33.3–50	0.002–0.03	Visual cortex	Rat (in vivo)	Demonstrated VEP elicited by focused transcranial ultrasonic stimulation in both normal and retinal degenerative rats.

* The resolution was quantified by the mean full width at half maximum (FWHM) of the focal plane. VEP: Visual-evoked potential. PRF: Pulse repetition frequency. RGCs: Retinal ganglion cells.
